# Low dose bisphenol A impairs spermatogenesis by suppressing reproductive hormone production and promoting germ cell apoptosis in adult rats

**DOI:** 10.7555/JBR.27.20120076

**Published:** 2012-12-21

**Authors:** Pengpeng Jin, Xiaoli Wang, Fei Chang, Yinyang Bai, Yingchun Li, Rong Zhou, Ling Chen

**Affiliations:** a State Key Laboratory of Reproductive Medicine, Nanjing Medical University, Nanjing, Jiangsu 210029, China;; b Department of Physiology, Nanjing Medical University, Nanjing, Jiangsu 210029, China;; c Department of Physiology, Wenzhou Medical College, Wenzhou, Zhejiang 325000, China.

**Keywords:** bisphenol A (BPA), spermatogenesis, apoptosis, testosterone

## Abstract

Bisphenol A (BPA), an estrogenic chemical, has been shown to reduce sperm count; however, the underlying mechanisms remain unknown. Herein, we show that oral administration of BPA (2 µg/kg) for consecutive 14 days in adult rats (BPA rats) significantly reduced the sperm count and the number of germ cells compared to controls. The serum levels of testosterone and follicle-stimulating hormone (FSH), as well as the level of *GnRH* mRNA in BPA rats were lower than those of control rats. Testosterone treatment could partially rescue the reduction of germ cells in BPA rats. Notably, the number of apoptotic germ cells was significantly increased in BPA rats, which was insensitive to testosterone. Furthermore, the levels of *Fas*, *FasL* and *caspase-3* mRNA in the testicle of BPA rats were increased in comparison with controls. These results indicate that exposure to a low dose of BPA impairs spermatogenesis through decreasing reproductive hormones and activating the Fas/FasL signaling pathway.

## INTRODUCTION

Bisphenol A (BPA) is an estrogenic compound that is widely used in the manufacture of polycarbonate plastics, which serve as containers for foods and beverages, as well as a constituent of dental sealants[1]. It is reported that 10-20 µg of BPA/can in canned food is leached from the lacquer lining[2]. In the saliva of patients who had been treated with a dental sealant, 20-30 µg of BPA/mL was detected[3]. The current reference dose [50 µg/kg/day (U.S. EPA 1993)] determined by the U.S. Environmental Protection Agency (EPA) is 1/1000 of the level that exerts the lowest adverse effect (LOAEL; 50 mg/kg/day). Although no evidence indicates that oral ingestion of BPA at typical environmental exposure level causes adverse effects, blood BPA levels in a group of pregnant women and their fetuses ranged from 0.3-18.9 and 0.2-9.2 ng/mL, respectively[4]. As BPA has been measured in several human populations, the effects of environmental doses of BPA on male fertility have recently attracted much attention. Multiple studies have shown that BPA, at doses even lower than the current reference or “safe” exposure limit for humans, can impair reproductive physiology and behavior in rodents[5]. A number of studies showed that the exposure of adult rats to environmental doses of BPA can reduce the sperm count and the efficiency of spermatogenesis[6],[7]. Adult male mice show significant reductions in testicular sperm counts, as well as in epididymal sperm counts, after exposure to 25 ng/kg BPA[8]. However, the mechanisms underlying BPA-reduced sperm count remain unknown.

Spermatogenesis is regulated by follicle-stimulating hormone (FSH) and testosterone released from the Leydig cells in response to luteinizing hormone (LH). In adult animals, androgen acting on Sertoli cells can promote and maintain the development of germ cells[9]. FSH stimulates spermatogenesis through increasing the number of spermatogonia and enhancing subsequent entry of these cells into meiosis[10]. FSH and testosterone have been reported to exert positive and overlapping effects on meiotic divisions and post-meiotic expression of a germ cell-specific gene. Earlier studies demonstrated that reduced levels of FSH or testosterone can affect spermatogenesis and reproductive function[11]. BPA has been reported to cause adverse effects on organs that are sensitive to estrogen or androgen by interfering with the interaction of endogenous reproductive hormones. Mendiola et al.[12] reported that in fertile men, exposure to a low level of BPA causes a modest decrease in testosterone level. Tohei et al.[13] reported that exposure of male adult rats to BPA decreased the plasma concentration of testosterone, but increased LH concentration.

To explore the molecular mechanisms of BPA-impaired spermatogenesis, we gave adult rats oral BPA at 2 µg/kg body weight per day (BPA rats) for 14 days, which corresponds to an environment level of BPA[14]. Ability of spermatogenesis, hormonal levels of the hypothalamic-pituitary-testicle (HPT) axis and testicle apoptotic index were compared between the control rats and BPA rats. Our results provide evidence that the low dose of BPA impairs spermatogenesis through not only reducing reproductive hormones but also inducing the apoptosis of germ cells.

## MATERIALS AND METHODS

### Experimental animals

The study protocol was approved by the Animal Care and Ethical Committee of Nanjing Medical University. All procedures were in accordance with the guidelines of the Institute for Laboratory Animal Research of the Nanjing Medical University. Adult male Sprague-Dawley rats (12-weeks-old, Oriental Bio Service Inc., Nanjing, Jiangsu, China)) were used throughout the study. Animal rooms were maintained on a 12:12 light-dark cycle starting at 7:00 am and kept at a temperature of 22-23°C. The animals had free access to food and tap water that were provided by glass bottles. To minimize BPA contamination through plastic cages, clean cages were lined with wood bedding. All efforts were made to minimize animal suffering and to reduce the number of animals used.

### Preparation of rat models

Rats were randomly divided into five groups (*n* = 10/group). There were three BPA-treated groups: one was single treatment with BPA; the other two groups of BPA rats were treated with testosterone propionate (TP) or vehicle, respectively. There were two control groups: in one group, rats were sacrificed at baseline (the baseline control group); the other was given vehicle in an identical manner (the experimental control group).

Oral administration of BPA: BPA (> 99% purity; Sigma, St. Louis, MO, USA) was diluted in olive oil at 150-200 µL. Rats were gavaged with BPA at 2 µg/kg body weight per day for consecutive 14 days. Control rats were treated with an equivalent volume of vehicle.

Subcutaneous (s.c.) injection of TP: TP (> 99% purity; Sigma) was dissolved in dimethylsulfoxide (DMSO) and then in 0.9% saline to a final concentration of 1.0% DMSO. TP was injected (s.c.) at a dose of 0.1 mg/rat per day as previously described[16]. Control and BPA rats were treated with an equivalent volume of vehicle.

### Epididymal sperm analysis

One epididymis was minced with fine scissors in 4 mL normal saline at 37°C and then filtered through one piece of gauze. The number of sperm was measured using a hemocytometer[8] under a light microscope (Olympus, Tokyo, Japan) at a magnification of ×40 and expressed as 10^7^ per gram of epididymal weight.

### Quantitative analysis of spermatogenesis

Testicular tissues were fixed in Bouin's fluid, and then were dehydrated through gradient alcohol, cleared in xylene and embedded in paraffin. Sections (5-µm-thick) were rehydrated, and stained with hematoxylin and eosin. Germ cells at stage VII of the seminiferous cycle, including type-A spermatogonia, preleptotine spermatocytes, mid-pachytene spermatocytes and step 7 spermatids in 10 round seminiferous tubules per testicle[17], were counted using a conventional light microscope (Olympus PD70) with a 100×objective. Type A spermatogonia and preleptotine spermatocytes mainly distribute in the basal side of seminiferous tubule (outfield). Type A spermatogonia have diffuse nucleus with fine-granulated chromatin. The preleptotene spermatocytes are characterized by thread-like clumps of chromatin. Mid-pachytene spermatocytes and step 7 spermatids lie close to the side of tubule lumen (infield). The mid-pachytene spermatocytes are characterized by large nucleus and mottled chromatin. The small step 7 spermatids lack heterochromatin.

### Measurement of hormones

For measurement of serum levels of LH, FSH and testosterone, blood samples were taken during the light phase 09:00-10:00 by jugular venipuncture. Serum (total 300 µL per rat) was separated by centrifugation at 4°C and stored at -80°C until assay. For measurement of intratesticular testosterone, testis was weighed and minced in 2 mL of 5% SDS in 0.5 N NaOH and incubated in a shaker bath with 100 rpm at 40°C for 4 hours. Steroids were extracted in 10 volumes of diethyl ether (Merck & Co Inc, Whitehouse Station, NJ, USA). The ether extract was allowed to evaporate overnight and reconstituted in 0.01 mol/L PBSG (0.01 M phosphate buffer, 0.1% gelatin, 0.15 mol/L NaCl, pH 7.4)[18]. LH, FSH and testosterone were measured using a radioimmunoassay (RIA) kit provided by the National Hormone and Peptide Program (Baltimore, MD, USA). The intra- and inter-assay coefficients of variation were 5.5% and 8.9% for LH, 4.3% and 10.3% for FSH, 6.2% and 7.4% for testosterone, respectively. Intratesticular testosterone was expressed as per gram of testicular tissue.

### Immunohistochemistry of GnRH

Rats were anesthetized with 10% chloral hydrate (400 mg/kg *i.p.*) and perfused intraventricularly with ice-cold PBS followed by 4% paraformaldehyde between 16:00-18:00. Brains were removed and post-fixed overnight in 4% paraformaldehyde, and then were transferred gradually into 15% and 30% sucrose until they settled. Every second section (40-µm-thick) through the whole preoptic area (from 0.36 mm anterior to 0.48 mm posterior to the bregma) was cut using a cryostat. The sections from the preoptic area were pre-incubated in blocking solution and then transferred in a mouse monoclonal anti-GnRH antibody (Chemicon International) for 24 h at 4°C with agitation. The sections were incubated with secondary goat anti-mouse antibody (Bioworld Technology Co., USA). Immunoreactivity was visualized with the standard avidin-biotin complex reaction with Ni-3,3-diaminobenzidine (DAB, Vector Laboratories). Sections were mounted on SuperFrost Plus slides (VWR Scientific, West Chester, PA., USA), air-dried, and dehydrated in gradient ethanol.

GnRH immunoreactive (GnRH^+^) cells in the preoptic area were counted using a conventional light microscope (Olympus PD70, Olympus, Japan) with a 40°C objective. The GnRH^+^ cells were counted in seven sections of the preoptic area. The GnRH^+^ cells per brain were summed to provide a single value. The values obtained from ten rats were averaged to provide each group value.

### TUNEL staining and quantification of apoptotic seminiferous tubules

Testicular tissues were fixed in Bouin's fluid at 4°C for 48 hours. The tissues were dehydrated through gradient alcohol and xylene, and then were embedded in paraffin. Apoptotic cells in the testicular tissues were examined by in situ terminal deoxynucleotidyl transferase-mediated digoxigenin-dUTP nick-end labeling (TUNEL) assay using an Apoptotic Detection Kit (Roche, Mannheim, Baden-Wuerttemberg. Germany). Sections (5-µm-thick) were treated with 1% H_2_O_2_ for 5 minutes to block endogenous peroxidase activity, and then subjected to 10 µg/mL proteinase K digestion for 15 minutes. The sections were incubated in TUNEL Mix (Roche) for 60 minutes in moist chamber at 37°C. After washing, the sections were incubated with horseradish peroxidase goat anti-biotin antibody at 37°C for 30 minutes. The cells with brown stained nuclei were observed as apoptotic cells using a conventional light microscope (Olympus PD70) with a 40× objective. Each cross-sectioned seminiferous tubule containing more than 3 TUNEL immunoreactive cells was counted as an apoptotic tubule as described by Li et al.[19]

### Real time-PCR (RT-PCR)

Rats were anesthetized with chloral hydrate (400 mg/kg, *i.p.*). Brains and testes were removed and immediately stored at -80°C until assayed. Brains were blocked and sections (50-µm-thick) were cut in the coronal plane using a cryostat. The preoptic area (POA) from the frozen slices was micro-dissected on dry ice. Total RNA was isolated from POA or testes using Trizol reagent kit (Invitrogen Life Technologies, Cergy Pontoise, France) according to the manufacturer's instructions. RNA (1 µg) was used to reverse transcribe using high-capacity cDNA of the reverse transcription kit RT (Applied Biosystems, Foster City, CA, USA) in accordance with the instructions. The primer sequences of *GnRH, GAPDH, AR, Fas, FasL* and *caspase-3* mRNA were designed according to the previous publications[20]–[22]. RT-PCR was performed using a Light Cycler Fast Start DNA Master SYBR Green I kit and an ABI Prism 7300 Sequence Detection System (Applied Biosystems) and relative expression of genes was determined using the 2-ΔΔCt method with normalization to GAPDH expression. Results were averaged from four sets of independent experiments (*n* = 10). The levels of GnRH, AR, Fas, FasL and *caspase*-*3* mRNA are expressed as percent of control value.

### Statistical analysis

Data were retrieved and processed with the software Micro-Cal Origin 6.1 (Micro-Cal Software Inc., Northampton, MA, USA). The group data was expressed as the mean±standard error (SE). Difference between two groups was evaluated using Student's *t* test. For one-variable experiments with more than 2 groups, significance of differences among means was evaluated using one-way ANOVA followed by Bonferroni's post-hoc tests. Statistical analysis was performed using State7 software (Stata Corp., College Station, TX, USA). Differences at *P* < 0.05 were considered statistically significant.

## RESULTS

### Influence of BPA on spermatogenesis in adult rats

**Fig. 1:**
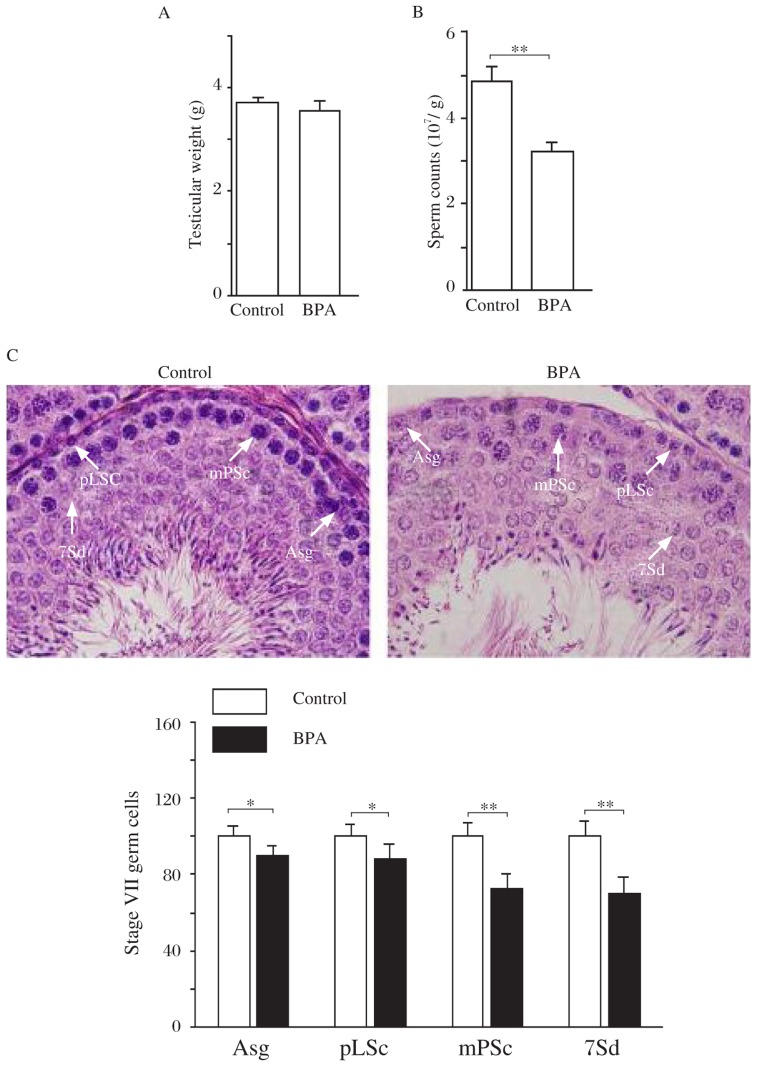
Influence of low dose BPA on spermatogenesis in adult rats. A: Mean value of both testicular weights in control and BPA rats. B: Sperm count in the epididymis. Bar graphs show the average number of sperm heads. **P < 0.01. C: Numbers of spermatogonia and spermatocytes at stage VII of the spermatogenic cycle in 100 seminiferous tubules. *P < 0.05, **P < 0.01. Type A spermatogonia (Asg), preleptotene spermatocytes (pLSc), mid-pachytene spermatocytes (mPSc) and step 7 spermatids (7Sd) are indicated by white arrows, respectively. Scale bar=10 µm.

The exposure of adult rats to 2 µg BPA/kg/day for 14 days (BPA rats) did not affect testes weight (*P* > 0.05, ***Fig 1A***). The sperm count was significantly reduced in the BPA rats compared to the control rats (sperm head count, *P* < 0.01, ***Fig 1B***). In comparison with the control rats, oral BPA significantly decreased the number of type A spermatogonia (*P* < 0.05), preleptotene spermatocytes (*P* < 0.05), mid-pachytene spermatocytes (*P* < 0.01) and step 7 spermatids (*P* < 0.01, ***Fig 1C***).

### Influence of BPA-reduced testosterone on spermatogenesis

**Fig. 2:**
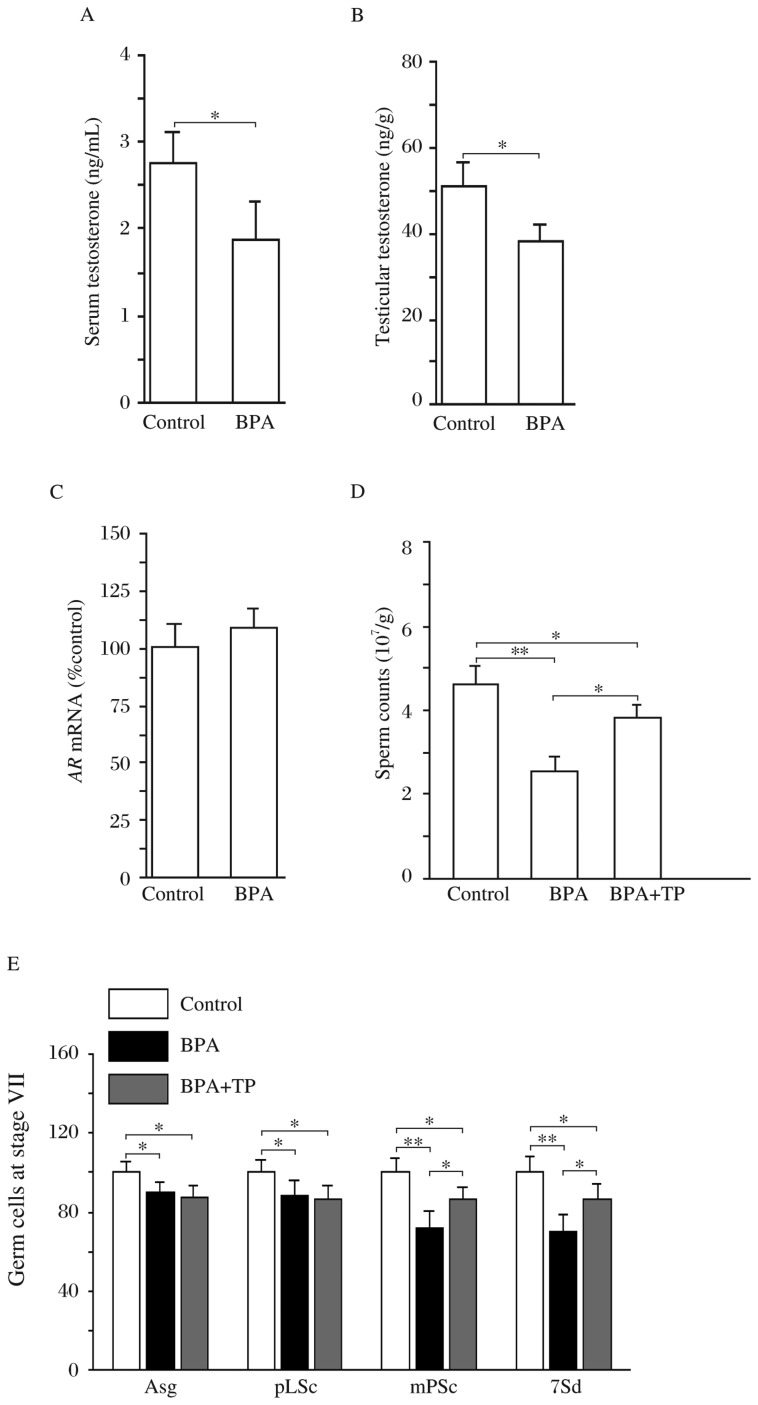
Influence of BPA-reduced testosterone on spermatogenesis. A and B: Levels of testosterone in serum and testicle. *P < 0.05. C: Level of AR mRNA in testes of control and BPA rats. D: Effects of testosterone replacement on reduction of sperm count in BPA rats. *P < 0.05 and ** *P* < 0.01 vs. control rats; ^#^*P* < 0.05 vs. BPA rats treated with vehicle by ANOVA analysis followed by Bonferroni's post-hoc test. E: Effects of testosterone replacement on the reduction of spermatogonia and spermatocytes in BPA rats. Bar graph shows relative values of type A spermatogonia (Asg), preleptotene spermatocytes (pLSc), mid-pachytene spermatocytes (mPSc) and step 7 spermatids (7Sd) in 100 seminiferous tubules. *P < 0.05 and **P < 0.01.

The levels of serum testosterone and intratesticular testosterone were lower in the BPA rats than those in the control rats (*P* < 0.05, ***Fig 2A&2B***). The level of *AR* mRNA in testes had no significant difference between the control and BPA rats (*P* > 0.05, ***Fig 2C***). To test whether low testosterone levels affected spermatogenesis, BPA rats were given TP s.c.. The results showed that TP partially rescued the reduction of germ count compared to vehicle-treated BPA rats (*P* < 0.05, ***Fig 2D***). Similarly, the reduction of mid-pachytene spermatocytes or step 7 spermatids in the BPA rats was attenuated by testosterone (*P* < 0.05, ***Fig 2E***). However, administration of TP in the BPA rats had no effect on the decrease in the number of type A spermatogonia and preleptotene spermatocytes (vs. vehicle-treated BPA rats, *P* > 0.05). The results indicate that the reduction in testosterone levels by BPA arrests the process of spermatocyte meiosis.

### Effects of BPA on pituitary gonadotropin and GnRH

**Fig. 3:**
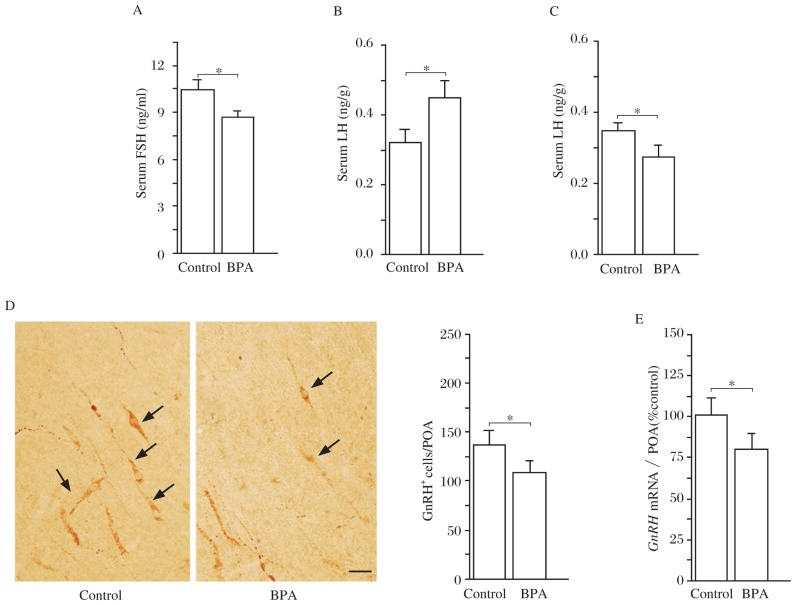
Effects of BPA on gonadotropic hormones and GnRH neurons in adult rats. A and B: Levels of serum LH and FSH. *P < 0.05. C: Following testosterone replacement, the level of LH was lower in the BPA rats than that in the control rats. *P < 0.05. D: Photographs of GnRH- immunohistochemistry in preoptic area (POA). Arrows indicate GnRH+ cells. Scale bar=50 µm. Bar graph shows mean number of GnRH+ cells. *P < 0.05. E: Relative level of *GnRH* mRNA in POA. *P < 0.05.

To investigate the mechanisms underlying BPA-reduced testosterone, we examined pituitary gonadotropin and GnRH. In comparison with the control rats, the serum level of FSH in the BPA rats was significantly reduced (*P* < 0.05, ***Fig 3A***), whereas LH level was slightly elevated (*P* < 0.05, ***Fig 3B***). Notably, the level of LH in the BPA rats treated with TP was lower than the control rats (*P* < 0.05, *n* = 10; ***Fig 3C***). In addition, the number of GnRH immunoreactive (GnRH+) cells (*P* < 0.05, ***Fig 3D***) and the level of GnRH mRNA in the preoptic area of the BPA rats (*P* < 0.05, *n* = 10; ***Fig 3E***) were significantly lower than those in the control rats. The results indicate that BPA reduces the levels of GnRH and pituitary gonadotropin, leading to reduced testosterone levels.

### Effects of BPA on apoptosis of germ cells in the testicle

**Fig. 4:**
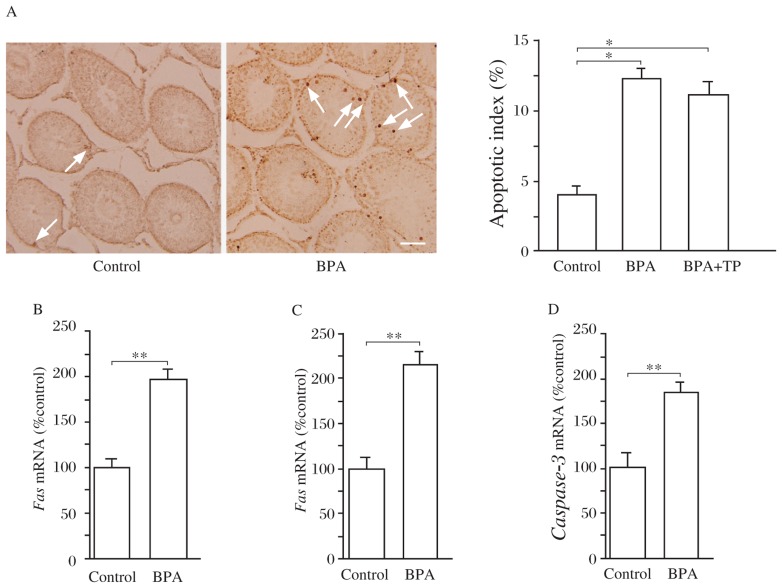
Effects of BPA on apoptosis of germ cells. A: Quantitative analysis of apoptotic cells in the testicle. The apoptotic index is expressed as percentage of apoptotic seminiferous tubule (containing more than three apoptotic germ cells). **P < 0.01. Arrows indicate TUNEL positive cells. Scale bar=100 µm. B-D: Expression of *Fas, FasL*, and *caspase*-*3* in the testes. Bar graphs show the relative levels of *Fas* mRNA (B), *FasL* mRNA (C) and the *caspase*-*3* mRNA (D) in the testes of the control rats and BPA rats. **P < 0.01.

To further explore the mechanisms of BPA-impaired spermatogenesis independent of testosterone replacement, we examined the apoptosis of germ cells. As shown in Figure 4A, TUNEL positive cells in the seminiferous tubule were significantly increased in the BPA rats compared to the control rats. The apoptotic index was higher in the BPA rats than that in the control rats (*P* < 0.01), which recalcitrant to testosterone replacement (*P* > 0.05, ***Fig 4A***). In addition, RT-PCR showed that the levels of Fas and FasL and caspase-3 mRNA in the testicle of the BPA rats was significantly increased compared to the control rats (*P* < 0.01, ***Fig 4B-D***). The results indicate that exposure to low dose of BPA can activate the Fas/FasL signaling pathway to induce apoptosis of germ cells.

## DISCUSSION

The present study provides evidence that exposure to low doses of BPA impairs spermatogenesis through reducing reproductive hormones to arrest meiosis of germ cells and activating the Fas/FasL pathway to induce the apoptosis of germ cells. Consistent with our results, Mendiola et al.[12] reported that exposure to environmental dose of BPA decreased the plasma level of testosterone in adult male rats. The biosynthesis or secretion of testosterone in Leydig cells is controlled by LH. Tohei et al.[13] reported that exposure of adult rats to BPA decreased the plasma concentration of testosterone, which was associated with an increase in the plasma concentration of LH. Similarly, our results showed that the testosterone level was reduced in the BPA rats, which was accompanied with an increase in LH levels. Because replacement of testosterone by the administration of TP could prevent increase in LH in BPA rats, it is conceivable that the testosterone-induced negative feedback is depressed in BPA rats, leading to increase in LH biosynthesis and secretion. On the other hand, Akingbemi et al.[14] found that oral administration of BPA (2.4 µg/kg/day) for 14 days could inhibit testosterone production of Leydig cells *in vitro* through decreasing the expression of steroidogenic enzyme 17α-hydroxylase/17-20 lyase[14] or increasing aromatase activity to enhance the reduction of testosterone[23]. Our results did not reveal changes in the serum level of estrogen (data not shown). In addition, we observed that the number of GnRH^+^ cells and the level of GnRH mRNA in the preoptic area were lower in the BPA rats than those in the control rats. Therefore, it is proposed that BPA reduces the biosynthesis and secretion of testosterone probably through inhibiting the function of GnRH neurons and decreasing the expression of steroidogenic enzymes.

Our results showed that oral BPA decreased the number of type A spermatogonia, preleptotene spermatocytes, mid-pachytene spermatocytes and step 7 spermatids. Declining testosterone level is accompanied by reduction of sperm concentration[24]. Mice lacking functional AR show severe disruption to spermatogenesis[25]. Testosterone plays a key role in the process of spermatocyte meiosis[26],[27]. Exogenous testosterone can increase the production of round spermatids from pachytene spermatocytes[28]. We observed that testosterone replacement in the BPA rats could rescue the reduction in the number of mid-pachytene spermatocytes and step 7 spermatids. In addition, Sertoli cells have been reported to act as a central regulator of testicular function and are essential for maintenance of spermatogenesis through direct interactions with developing germ cells. It is clear that regulation of Sertoli cell proliferation and activity in the adult animal is crucial for normal adult fertility. Previous studies have shown that FSH can regulate Sertoli cell function. Initial evidence comes from the demonstration that FSH can maintain spermatogenesis in hypophysectomized rats, and it is subsequently shown that FSH can stimulate aspects of Sertoli cell function. Reduction of FSH causes the diminution of germ cells at stage VII of spermatogenic cycle[11]. The increase of FSH can attenuate the degeneration of type A spermatogonia, while the reduction of FSH may promote degeneration of type A spermatogonia[11]. FSH β-subunit knockout mice and FSH receptor knockout mice show the reduction in the number of germ cells and sperm quality[29]. Thus, it is indicated that reduction in testosterone and FSH levels following exposure to BPA potentially arrests the meiosis of spermatocytes, which leads to the reduction in the number of germ cells.

Another important finding of this study is that exposure of adult male rats to a low dose of BPA induced the apoptosis of germ cells, which was insensitive to TP. The Fas-signaling system is considered to be a key regulator of germ cell apoptosis during development and after testicular insults[30],[31]. Our results showed that BPA exposure increased the levels of Fas, FasL and caspase-3 mRNA in the testes. Engagement of Fas by FasL causes the clustering of Fas-associated protein with death domain (FADD) and the activation of caspase-8. Caspase-3, as a main final common executor of apoptosis, is responsible for the cleavage of the key cellular proteins leading to cell apoptosis[32]. Actually, Li et al.[19] reported that high expression of Fas and active caspase-3 were localized at the apoptotic germ cells in mice exposed to oral BPA at puberty. Thus, our findings indicate that low-dose BPA-induced apoptosis of germ cells might be the result, at least in part, of the Fas-mediated death receptor pathway[30]. There is, however, a conflicting report[19] describing that high dose BPA (480 and 960 mg/kg/day) could induce the apoptosis of germ cell and the expressions of Fas, FasL and active caspase-3 in the testes, but BPA at the dose of 160 mg/kg/day could not. By contrast, pubertal exposure of male mice to BPA (160 mg/kg) induces germ cell apoptosis in the testes and the Fas/FasL signaling pathway[33]. Sakaue et al.[7] found that the impact of BPA on spermatogenesis was not aggravated in a dose dependent manner over a BPA dose of 20 µg/kg, pointing out that sperm numbers do not decrease further with an increase in BPA dose. It is well documented that low dose of BPA mainly binds to estrogen receptors, while high dose BPA interacts with AR and thyroid hormone receptors. Hatef et al.[34] consider that BPA may exert both anti-androgenic and estrogenic effects, depending on concentration, to impair sperm production. Thus, the discrepancy regarding the apoptosis of germ cell and activation of the Fas/FasL pathway induced by high and low doses of BPA may be due to differences in the molecular mechanisms of BPA actions. A recent in vitro study has revealed the cytotoxicity of low dose BPA through the up-regulation of Bax and the down-regulation of Bcl-2[35]. Additionally, BPA-induced apoptotic cell death is known to be through calcium-mediated oxidative stress in HT-22 cells[36] and decreasing ERK and Akt phosphorylation in acute myeloid leukemia cells[37]. Moreover, BPA-induced impairment of Sertoli cells has been reported by inhibiting endoplasmic reticulum Ca^2+^ homeostasis[38],[39] and the ectoplasmic specialization between Sertoli cells and spermatids[40].

In summary, a number of studies point out that the adult exposure to environmental doses of BPA can reduce sperm production and efficiency of spermatogenesis[6],[7]. The present study provides evidence that exposure of adult rats to low doses of BPA impairs spermatogenesis through decreasing reproductive hormones and activating the Fas/FasL pathway in testicular tissues.
